# Extreme Edge Computing for Secure and Private Multimodal Biometric Identification in Intelligent IoT Systems

**DOI:** 10.3390/s26123756

**Published:** 2026-06-12

**Authors:** José Antonio de la Torre, Fernando Rincón, Soledad Escolar, Antonio Caruso, Julián Caba, Jesús Barba

**Affiliations:** 1 Technology and Information Systems Department, School of Computer Science, University of Castilla-La Mancha, 13071 Ciudad Real, Spain; fernando.rincon@uclm.es (F.R.); soledad.escolar@uclm.es (S.E.); julian.caba@uclm.es (J.C.); jesus.barba@uclm.es (J.B.); 2 Department of Mathematics and Physics ‘Ennio De Giorgi’, Palazzo Fiorini, Campus Ecotekne, 73100 Lecce, Italy; antonio.caruso@unisalento.it

**Keywords:** TinyML, edge AI, multimodal biometrics, Compute Continuum, Internet of Things (IoT), embedded systems, privacy-by-design, facial recognition, voice recognition, energy efficiency

## Abstract

The exponential growth of Internet of Things (IoT) ecosystems is driving a paradigm shift from centralized cloud computing towards decentralized architectures to mitigate latency and bandwidth constraints. While edge computing addresses some of these challenges, data transmission to local gateways still raises critical security and privacy concerns. This study explores the Compute Continuum by pushing intelligence to the extreme edge using TinyML. We propose a secure, privacy-preserving multimodal biometric authentication system designed for resource-constrained embedded devices. Our solution implements a hierarchical processing chain: an ultra-lightweight person-detection filter acts as an intelligent wake-up mechanism, followed by robust facial and voice authentication modules. Operating as a strict hierarchical pipeline, the system achieves a combined False Acceptance Rate (FAR) of just 0.12%. Experimental results on an ESP32 microcontroller demonstrate exceptional energy efficiency, requiring only 0.15 J per inference cycle. This allows the system to operate autonomously for over 39 h of continuous inference on a standard 600 mAh battery, proving the viability of standalone, privacy-by-design biometric sensors in intelligent IoT environments.

## 1. Introduction

Recent advancements in computing power and communication technologies, coupled with significant reductions in hardware costs, have exponentially expanded the application domains of embedded systems. This technological shift has driven the transition from isolated microcontrollers to interconnected, intelligent sensing nodes. By leveraging sophisticated algorithmic processing directly at the edge, these modern systems can perceive and dynamically respond to their environments with unprecedented autonomy. Consequently, smart embedded solutions are now ubiquitous across diverse sectors, including healthcare [1,2,3], industrial automation [4,5], smart cities [6,7], and smart home environments [8,9]. Highlighting this ubiquitous deployment, machine-to-machine (M2M) connections are projected to grow by 12.7% by 2029 [10], underlining the critical and growing need for scalable IoT architectures.

Concurrent with the expansion of IoT, the integration of Machine Learning (ML) has fundamentally redefined sensor capabilities. As demonstrated in recent literature [11], there is a powerful synergy between ML and IoT: smart sensors generate the vast datasets required to train robust models, while these models, in turn, enhance embedded devices with advanced analytical intelligence.

This integration has relied on cloud-centric architectures, where IoT nodes merely act as data transmitters, offloading raw sensory data to remote, high-performance servers for inference. While effective in smaller-scale deployments, the exponential proliferation of connected devices has exposed critical bottlenecks in this model, primarily network congestion, high transmission latencies, and excessive bandwidth consumption. Beyond these operational constraints, transmitting raw data to centralized cloud servers introduces critical privacy and security vulnerabilities. These risks are critically amplified when dealing with highly sensitive personal information, making decentralized processing an absolute necessity for applications such as the multimodal biometric authentication systems addressed in this study [12,13].

To mitigate these challenges, the paradigm of edge computing has emerged, decentralizing computation by moving it closer to the data source [14]. While edge computing utilizes local gateways with greater processing capabilities than the sensing nodes themselves, thus improving privacy and reducing latency compared to cloud servers, it still relies on continuous data transmission between the sensor and the local edge device. To eliminate this communication overhead entirely, a further paradigm shift is required. As proposed by Warden and Situnayake, the data processing pipeline can be pushed directly onto the sensing node itself [11]. This approach, which advocates for executing machine learning algorithms natively on highly constrained embedded devices at the extreme edge, is termed *TinyML*.

TinyML represents the culmination of the processing architecture often conceptualized as the *Compute Continuum* [15,16]. By executing machine learning models directly on the sensing node, this paradigm not only drastically reduces communication latency but also establishes “privacy by design”. This is crucial for sensitive biometric data, as transmitting it to external devices creates significant privacy risks [17]. Past incidents show that relying on cloud-based biometric authentication introduces notable security risks [18].

Addressing these critical challenges, this study leverages TinyML to develop a secure, privacy-preserving multimodal biometric system operating entirely at the extreme edge. We propose three optimized ML models: facial authentication, voice authentication, and a primary person-detection filter that acts as an intelligent wake-up mechanism to activate the system only upon human presence. The complete solution is deployed on a cost-effective, resource-constrained ESP32 microcontroller (Espressif Systems, Shanghai, China). Demonstrating exceptional energy efficiency, the prototype achieves 38 h of uninterrupted, continuous inference powered by a standard 600 mAh battery.

It is important to note that the proposed extreme-edge biometric inference acts as a localized factor of user verification. This on-device intelligence is entirely orthogonal to, and can be seamlessly integrated with, standard cryptographic IoT authentication protocols such as FIDO2 or FDO (FIDO Device Onboard) for secure network credential exchange.

To summarize, the main contributions of this work are as follows:

- The design and implementation of a fully autonomous, multimodal biometric authentication pipeline (person, voice, and face) optimized strictly for extreme-edge microcontrollers (TinyML).
- The design and optimization of a hierarchical, early-exit filtering framework that cascades three distinct deep learning models natively on a resource-constrained microcontroller, achieving drastic energy reductions through conditional verification states.
- Empirical validation of the system on a commercial off-the-shelf ESP32 microcontroller, demonstrating an ultra-low energy footprint of 0.15 J per inference cycle, enabling months of autonomous operation.
- A “privacy-by-design” architecture that completely eliminates the need for raw data transmission to cloud servers, mitigating network latency and safeguarding sensitive biometric information.

The remainder of this paper is structured as follows. First, an overview of key concepts in the field of biometrics and TinyML is presented in Section 2. This is followed by an analysis of related work in Section 3, which examines biometrics in embedded systems and the Internet of Things. Section 4 provides detailed descriptions of the biometrics models implemented and the integration strategy employed. In Section 5 we show the results obtained after testing each model. These findings are analyzed and contextualized within the current state of the art in Section 6. Finally, in Section 7 the lines of work identified in this research in the area of TinyML and embedded biometrics are described.

## 2. Background

### 2.1. TinyML

Banbury et al. [19] identify the core challenges and opportunities of TinyML, categorizing them into four primary domains: (1) low power budgets, (2) low memory capacity, (3) hardware heterogeneity, and (4) software heterogeneity. Based on these constraints, they classify typical use cases according to the input type and the underlying machine learning model.

The constraints identified by Banbury et al. have driven substantial research in subsequent years. For instance, the study by Xu et al. addresses the critical challenge of minimizing the power consumption (1) of neural networks [20]. In their work, the authors achieved a performance of 30 frames per second (FPS) for object detection with a power budget of only 160 mW. This was accomplished using a hardware–software co-design methodology, a highly effective approach within the TinyML paradigm as described by Capogrosso et al. [21]. First, they defined a custom neural network, named Etinynet, tailored specifically for object detection, which possesses a memory footprint of just 477 KiB. To further minimize power consumption, they designed an Application-Specific Integrated Circuit (ASIC) to execute the computations directly on hardware via a coprocessor. While this hardware-centric approach is highly promising for new designs, it requires replacing existing deployed devices, rendering it economically unfeasible for brownfield industrial scenarios [22].

To address low memory capacity (2), recent advances have focused on both software optimizations and architectural improvements in machine learning models [21]. A prominent strategy involves reducing the mathematical complexity and overall size of the network. Han et al. proposed a comprehensive three-stage compression pipeline: pruning, trained quantization, and Huffman coding [23]. When applied to a standard Convolutional Neural Network (CNN) such as VGG-16, this methodology reduces the model size from 552 MiB to 11.3 MiB, a 46-fold compression. The process begins with standard training, followed by pruning connections with weights below a predefined threshold. The subsequent step, quantization and weight sharing, is achieved by identifying weight clusters. Finally, the remaining weights are encoded using the Huffman algorithm, which yields an additional 30% reduction in the network’s storage footprint.

To overcome the remaining challenges of hardware and software heterogeneity (3, 4) identified in [19], the industry has developed robust interpreters and frameworks for edge deployment, most notably TensorFlow Lite [24]. These interpreters act as an abstraction layer between the high-level model definition and its low-level implementation, enabling highly efficient execution that leverages the specific hardware capabilities of diverse microcontrollers. More recently, industrial platforms such as Edge Impulse [25] have emerged to address both heterogeneity challenges simultaneously. By automating the compilation and deployment processes across disparate hardware targets, these platforms simplify what is increasingly known in the literature as TinyMLOps [26].

### 2.2. Biometrics

The term “biometrics” is defined as a set of technologies that use physical or behavioral characteristics to uniquely identify an individual [27,28]. Modern biometric systems rely on a synergy between physical sensors and advanced processing algorithms. Today, a vast array of sensors can capture these unique characteristics. For instance, an imaging sensor integrated into a smart building or an intelligent IoT gateway can capture physiological patterns that are subsequently processed to extract a unique user footprint. While biometric algorithms have been researched for decades, the recent explosion of machine learning and deep learning techniques has led to unprecedented enhancements in their accuracy and reliability [29,30].

Among the most commonly used human traits are ocular, facial, vascular, and fingerprint biometrics [31], with facial and fingerprint modalities currently dominating widespread deployment [32]. The underlying processing algorithms generally fall into two categories: classical approaches relying on manual feature engineering, and data-driven approaches using large-scale neural networks. The latter utilize extensive datasets containing positive and negative examples to train sophisticated models capable of discovering highly complex, non-linear patterns within the sensor data [33].

Integrating the ubiquitous data-collection capabilities of IoT sensor networks with advanced biometric analysis offers promising potential for smart environments. However, this convergence introduces critical challenges, primarily concerning the secure management of highly sensitive personal data on devices with severely constrained computational resources [34,35]. While some authors have proposed cloud computing as the solution to offload processing constraints [36,37], this architecture inherently compromises data privacy by transmitting sensitive user features outside of local control. Beyond privacy concerns, centralized cloud infrastructures present single points of failure. High-profile incidents, such as the global Facebook outage caused by a BGP configuration error where employees lost physical access to their facilities [38], underscore the critical fragility of cloud-dependent physical security. Consequently, alternative paradigms are urgently needed to foster trust and reliability in biometric IoT systems.

In this context, TinyML presents a compelling opportunity. By embedding inference capabilities directly onto the end-node sensors, TinyML allows biometric analysis to be performed entirely at the edge. This “privacy by design” approach eliminates the vulnerabilities associated with data transmission and external cloud reliance, ultimately making intelligent IoT systems more resilient, secure, and capable of autonomous user authentication.

## 3. Related Work

The theoretical foundations of multimodal biometrics have been extensively studied over the past two decades. Early foundational works, such as the comprehensive overview by Ross and Jain [39], established the critical advantages of combining multiple biometric traits, such as face and voice, to overcome the limitations of unimodal systems, including noisy data, intra-class variations, and spoof attacks. These early works laid the groundwork for fusion strategies at various levels (sensor, feature, and decision). However, their implementations were historically bound to traditional, high-performance computing environments, highlighting the need for the extreme edge approach proposed in this paper to ensure privacy and scalability in modern IoT systems.

Kocacinar et al. [40] proposed the use of lightweight Convolutional Neural Networks (CNNs) for detecting the proper use of masks during the COVID-19 pandemic. While the authors achieved high accuracy levels (90.40%) using the initial 23 layers of MobileNet combined with custom dense layers, the implementation was deployed on an Android smartphone via TensorFlow Lite. Smartphones possess vast computational resources and memory compared to typical IoT sensing nodes. Furthermore, the authors do not specify the optimization techniques employed, such as quantization, weight clustering, or pruning, which are critical for true extreme-edge deployment.

Huang et al. [41] examined an edge-based access control system for smart buildings to mitigate the high latency traditionally associated with cloud solutions, such as the approach proposed by Wei et al. [36]. To address this, the authors utilized a “computation module” at the edge, employing the FaceNet model [42] with triplet loss to calculate distinct facial embeddings. For the hardware implementation, they used an ESP32 microcontroller strictly for image capture and offloaded the processing to a Huawei P30 smartphone. Although this approach successfully reduced latency by 49% (down to 70 ms) compared to cloud solutions, the ESP32 merely acts as a data transmitter. Because it relies on continuous local connectivity to a high-performance edge gateway (the smartphone) rather than performing inference at the sensing node itself, it cannot be classified as a true TinyML solution.

Similarly, Zhang et al. [43] developed a voice biometric system for Android mobile devices utilizing Gaussian Mixture Models (GMM) to cluster Mel-frequency cepstral coefficients (MFCC) extracted from audio samples. They reported a robust accuracy of 89% and a processing latency between 210 and 320 ms. Despite these favorable metrics, the system is once again constrained to high-tier mobile devices, and the study lacks critical implementation details regarding the model’s footprint and specific hardware constraints.

Addressing audio processing on strictly constrained hardware, Vitolo et al. [44] focused on format conversion (PDM to PCM) for MEMS transducer signals, intended as a precursor stage for Key Word Spotting (KWS) in IoT devices. The authors replaced traditional algorithms, such as Cascaded-Integrator-Comb filters [45], with a custom neural network based on two 8-bit quantized CNN layers. For training, they implemented a custom loss function analogous to Mean Absolute Error (MAE) based on the Fast Fourier Transform (FFT), as shown in (1). This metric takes each ground truth and predicted FFT ($Y_{i}$ and ${\hat{Y}}_{i}$) and measures the mean difference. While this work successfully demonstrated the potential of adapting neural networks to embedded devices, achieving 89% accuracy with micro-joule energy consumption, the final implementation relies on a custom-designed ASIC using TSMC manufacturing technology. Although highly energy-efficient, the requirement for custom silicon drastically limits its flexibility and scalability compared to software-based TinyML deployments on commercial off-the-shelf (COTS) microcontrollers.

Recent studies continue to explore face and voice fusion using modern machine learning architectures. For instance, Alharbi and Alshanbari [46] developed a multimodal system utilizing FaceNet for facial recognition and Gaussian Mixture Models (GMM) for voice, achieving enhanced accuracy and a low Equal Error Rate through score-level fusion. Similarly, Byahatti and Shettar [47] proposed fusion strategies for face and voice cues to mitigate the vulnerabilities of unimodal systems. While these approaches achieve high accuracy, they rely on computationally intensive models (like FaceNet) that require standard desktop or server-grade hardware, making them incompatible with the extreme edge processing and tight memory constraints targeted in our work.

(1) $${\mathrm{FFT}}_{\mathrm{MAE}}=\frac{1}{n}\sum_{i=0}^{n}∥\mathrm{FFT}\left(Y_{i}\right)-\left|\mathrm{FFT}({\hat{Y}}_{i})\right|∥$$

In their study, Alaslani and Elrefaei [48] presented a hybrid approach combining a Convolutional Neural Network (CNN) and a Support Vector Machine (SVM) for iris recognition. They utilized a pre-trained AlexNet model, extracting feature maps from the intermediate layers to feed into the SVM. While they reported high accuracy (ranging from 89% to 100% across different datasets) and an inference latency between 60 and 90 ms, the system was executed on an Intel Core i5 processor. This reliance on desktop-grade hardware inherently limits its applicability in true embedded sensing scenarios.

Similarly, Al-Waisy et al. [49] proposed a deep learning-based multimodal biometric system. Although their feature extraction and ranking-level fusion methodology share conceptual similarities with our approach, their implementation targeted high-performance computing infrastructure, specifically an Intel Xeon E5 processor. They achieved an impressive precision of 99.82% with a latency of 620 ms, but such massive computational demands are completely incompatible with energy-efficient edge sensors.

Addressing the IoT domain specifically, Umer et al. [50] proposed a multimodal biometric recognition framework for connected devices. Their work provides a valuable analysis of various fusion techniques—including Borda count, ranking-level, and highest-rank fusion—alongside the use of cancellable biometrics to enhance privacy. However, the authors explicitly note that their solution was merely simulated using MATLAB 2016b on an Intel Core i5 processor, rather than being deployed and validated on physical, resource-constrained IoT nodes.

Overall, the literature focusing strictly on embedded or TinyML-based biometrics remains remarkably limited, primarily due to the relative infancy of the paradigm despite its rapid advancements. Furthermore, our review highlights a prevalent discrepancy in the field: numerous studies erroneously classify mobile phones as resource-constrained IoT devices. Modern smartphones, however, possess computational capabilities rivaling desktop computers, featuring 8 to 16 GiB of RAM, multi-core processors, and graphical processing units capable of teraflops (TFLOPS) of performance. Consequently, they cannot be equated with genuine extreme-edge IoT systems, such as microcontrollers lacking Memory Management Units (MMUs) or the capacity to run full operating systems. Table 1 summarizes the primary works reviewed in this section, emphasizing this severe hardware disparity. It is evident that the vast majority of deep learning-based biometric research relies on hardware platforms with orders of magnitude more resources than those targeted in this study. In contrast, our work demonstrates the feasibility of implementing robust multimodal biometric models on a strictly constrained edge device featuring merely 4 MiB of RAM and an 80–240 MHz processor.

## 4. Proposed Method

This section presents the design and implementation of an efficient multimodal biometric system tailored for resource-constrained embedded environments. The proposed architecture employs a hierarchical filtering strategy, integrating a preliminary person-detection module followed by voice and facial authentication stages. The final prototype was deployed on an ESP32 microcontroller, featuring dual Xtensa 32-bit LX6 cores, 8 MiB of RAM, and 4 MiB of Flash memory. Table 2 summarizes the specifications of the commercial development boards evaluated for this study. The ESP32 development kit was selected due to its optimal balance of low cost, superior RAM capacity compared to the alternative microcontrollers under consideration, and extensive external connectivity options.

**Figure 1:**
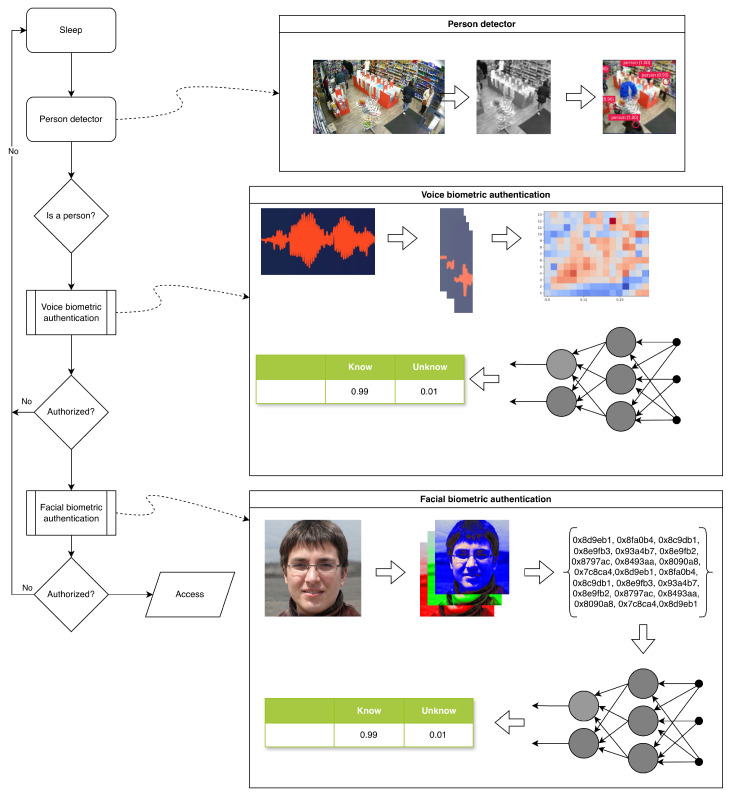
Overview diagram of proposed system.

Figure 1 depicts the architecture of the proposed solution, which includes three sequential detection modules: person, voice, and facial recognition. The initial stage acts as an intelligent visual wake-up mechanism based on person detection. While traditional systems often employ active sensors like PIR or radar to minimize continuous processing [52,53], such sensors merely detect motion or generic obstacles rather than specifically identifying human presence. Implementing an initial human-detection layer not only conserves computational resources by preventing unnecessary biometric processing but also mitigates potential presentation attacks. Upon confirming human presence, the system transitions to an active state to evaluate voice biometrics, determining initial authorization. Finally, a definitive verification is conducted via facial authentication. In this prototype, the modalities operate as a strict hierarchical filter; however, integrating more complex probabilistic fusion techniques, such as those described in [54,55], remains a promising avenue for future research.

### 4.1. Person Detection Module

As illustrated in Figure 1, the person detection module serves as the foundational gatekeeper of the system. Currently, the module’s output is binary, indicating solely the presence or absence of a person within the frame. However, because the underlying architecture utilizes a fine-tuned FOMO (Faster Objects, More Objects) model [56], it inherently calculates the spatial centroid of the detected object. Future iterations could leverage this spatial data to dynamically adjust capture parameters or guide the user into optimal alignment for subsequent biometric stages, though at the cost of increased processing complexity.

#### 4.1.1. Capture and Preprocessing

The hardware prototype integrates an OmniVision OV5640 CMOS sensor (OmniVision Technologies, Santa Clara, CA, USA). This 5-megapixel camera supports resolutions up to 2592 × 1944 pixels; its primary technical specifications are detailed in Table 3.

Given the tight memory and processing constraints of the ESP32, the raw input resolution must be drastically downscaled prior to inference. During the initial preprocessing stage, the image resolution is reduced from 640 × 480 to 96 × 96 pixels. Subsequently, the RGB image is converted to grayscale following the ITU-R BT.601-2 standard. This transformation calculates the luminance $Y_{i}$ for each pixel by applying a weighted sum to the respective color channels, utilizing optimized bitwise operations as described in (2).

(2) $$\begin{array}{cc} Y_{i} & =0.299\cdot \left(\frac{{\mathrm{pixel}}_{i}\gg 16\;\&\;0xFF}{255}\right)\\ \; & \;+0.587\cdot \left(\frac{{\mathrm{pixel}}_{i}\gg 8\;\&\;0xFF}{255}\right)\\ \; & \;+0.114\cdot \left(\frac{{\mathrm{pixel}}_{i}\;\&\;0xFF}{255}\right) \end{array}$$

#### 4.1.2. Proposed Model

The person-detection model is based on FOMO, an architecture introduced by Edge Impulse as a highly constrained alternative to traditional object detection networks like YOLO. FOMO prioritizes ultra-low latency and minimal memory footprint over pixel-perfect bounding box accuracy, making it ideal for microcontrollers. Unlike YOLO, which predicts complex bounding boxes, FOMO divides the input image into a configurable grid and predicts the presence of a target class centroid within each cell using a lightweight MobileNetV2 backbone [57]. While this grid-based approach can struggle if multiple overlapping objects occupy the same cell, it is highly effective for determining general human presence in an access-control scenario. The computational efficiency of FOMO compared to YOLO variants has been demonstrated in recent literature, such as the study by Silva et al. [58] detailed in Table 4.

The model was trained using the Adam optimizer [59]. The specific hyperparameters, including an initial learning rate of 0.001 and a training duration of 100 epochs, were determined empirically during the validation phase. The learning rate of 0.001 was selected as it is the standard and mathematically recommended starting value for the Adam algorithm to ensure stable gradient updates without overshooting the local minima. The training duration, 100 epochs provided sufficient iterations for the training and validation loss curves to converge.

#### 4.1.3. Dataset

**Figure 2:**
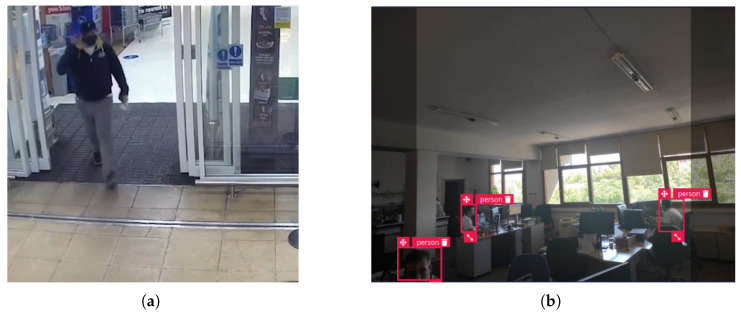
Training dataset examples for the person detection module: (**a**) sample from the public Kaggle human detection dataset; (**b**) manual labeling process of human subjects in the custom laboratory environment dataset using bounding boxes.

The dataset collected for this module combines publicly accessible data with custom images captured in the exact laboratory environment where the prototype operates. The custom subset comprises 200 RGB images (640 × 480 resolution, JPEG format) captured with the integrated OV5640 sensor to ensure the model adapts to the specific optical characteristics of the hardware. To ensure robust generalization and augment the training volume, a public dataset from Kaggle [60] (available under a Creative Commons license) was incorporated. This supplementary dataset features diverse indoor and outdoor CCTV captures, as illustrated in Figure 2a. Both datasets were labeled with bounding boxes to indicate human presence, Figure 2b, and subsequently split into an 80% training and 20% validation ratio.

### 4.2. Voice Biometric Module

Following the confirmation of human presence, the system activates the voice biometric module. This stage acts as the primary authorization filter, leveraging the built-in acoustic transducer to distinguish between the authorized user and unauthorized individuals or background noise (see Figure 1).

#### 4.2.1. Capture and Preprocessing

The audio signal is captured using an integrated MP34DT05 MEMS microphone (STMicroelectronics, Geneva, Switzerland); its primary specifications are detailed in Table 5. The sensor inherently outputs a Pulse Density Modulation (PDM) signal. To facilitate digital signal processing, this stream is converted internally by the microcontroller’s peripheral interface into standard Pulse Code Modulation (PCM) format.

For feature extraction, we opted for Mel-Frequency Cepstral Coefficients (MFCCs). While end-to-end deep neural networks can technically extract features directly from raw audio streams, they impose prohibitive memory and computational burdens on extreme-edge microcontrollers. Conversely, MFCCs offer a highly efficient, well-established feature engineering method that captures the critical phonetic characteristics of speech with minimal resource overhead, as demonstrated in similar constrained applications [61,62,63,64]. The PCM audio undergoes a standard preprocessing pipeline: the signal is divided into overlapping frames (framing and windowing), transformed into the frequency domain via the Fast Fourier Transform (FFT), and subsequently passed through Mel-scale filter banks. The specific hyperparameters tuned for optimal speaker authentication in this prototype are detailed in Table 6.

#### 4.2.2. Proposed Model

The extracted MFCC feature matrix is fed into a custom, lightweight 1D Convolutional Neural Network (CNN) tailored specifically for this architecture (see Table 7). Unlike the vision modules (facial recognition and person detection), this network was trained entirely from scratch without pre-trained weights.

Crucially, a Gaussian Noise layer was incorporated at the input. It is important to note that this layer functions strictly as a data augmentation and regularization technique, active only during the training phase. By artificially injecting noise into the MFCC features during training, the network is forced to learn robust representations, effectively simulating electrical transducer noise and unpredictable acoustic environmental variations [65,66]. This prevents the model from overfitting to the optimal laboratory recordings and ensures reliable generalization during real-world inference. The final layer employs a Softmax activation to output probabilities for three distinct classes: “authorized”, “background”, and “unknown/unauthorized”.

The network was trained using categorical cross-entropy loss with the Adam optimizer (learning rate of 0.001). Consistent with the established methodology, the dataset was split into an 80%/20% training–validation ratio.

The network was trained using cross-entropy loss with the Adam optimizer and a learning rate of 0.001. As in the other modules, the dataset was divided into 80% for training and 20% for validation.

#### 4.2.3. Dataset

In realistic personalized IoT environments, such as a smart lock or a customized smart home sensor, expecting end-users to provide thousands of voice samples for initial system enrollment is entirely impractical. Therefore, this module was deliberately designed and evaluated under a constrained, localized “few-shot” enrollment scenario.

The dataset for the “authorized” class consists of merely 100 audio captures of the target user speaking two specific passphrase commands (“Hi, I am [Name]” and “Open”). This approach is analogous to practical enrollment protocols proposed in recent literature [67]. All authorized samples were recorded at 16 kHz using the exact MP34DT05 hardware utilized for inference to ensure absolute sensor consistency. The “background” class comprises ambient noise captured in a standard office environment. Finally, the “unknown/unauthorized” class utilizes segments of human speech extracted and processed from open-source video repositories. By restricting the authorized training data to a realistic user-enrollment size, we demonstrate the genuine viability of deploying personalized biometrics on extreme-edge hardware.

### 4.3. Facial Biometric Module

The final module in the proposed hierarchy of biometric filters is the facial filter. This module is designed to identify individuals who have successfully passed the preceding filters (person detection and voice authentication). For this module, the same capture device is employed as for person detection, namely the OV5640 camera, in conjunction with a MobileNet V2 based neural network that has been specifically trained for facial authentication.

#### 4.3.1. Capture and Preprocessing

This module repurposes the same OV5640 CMOS camera utilized for the initial person-detection phase. However, unlike the preceding stages that utilize grayscale conversion to conserve memory, facial recognition relies heavily on complex feature extraction where chromatic information is highly valuable. Consequently, the initial 640 × 480 RGB capture is cropped and downscaled directly to 96 × 96 pixels while being strictly maintained in its native RGB color space. This results in a (96, 96, 3) input tensor, which preserves essential color features and ensures absolute structural compatibility with the pre-trained MobileNet neural network backbone.

#### 4.3.2. Proposed Model

Deploying state-of-the-art facial recognition architectures from scratch remains prohibitively expensive for an ESP32. To overcome this, we implemented an optimized Transfer Learning methodology utilizing a MobileNet backbone [57]. MobileNet models introduced Depthwise Separable Convolutions, which drastically reduce the mathematical complexity and parameter count compared to traditional CNNs. To further constrain the model size for extreme-edge deployment, we utilized a specific MobileNet architecture initialized with a width multiplier (α) of 0.25. This hyperparameter proportionally thins the network, significantly reducing the memory footprint while retaining the robust feature-extraction capabilities learned from large-scale pre-training. As detailed in Table 8, the top layers of the pre-trained base model were truncated and replaced with a custom, lightweight classification head. This head comprises a Reshape layer, a Dropout layer (rate = 0.1) to mitigate overfitting on the localized dataset, a Flatten layer, and a final Dense layer with Softmax activation to classify the input into three categories: “authorized”, “background”, and “unknown/unauthorized”.

To maximize both accuracy and efficiency, the dataset was partitioned into an 80% training and 20% validation split. The training process was executed in two distinct stages utilizing categorical cross-entropy as the loss function. The initial training phase was conducted over 150 epochs using the Adam optimizer [59] with a learning rate of 0.0005, keeping the entire MobileNet base frozen to allow the custom Dense layer to converge. Subsequently, a fine-tuning phase was performed over 10 epochs. During this stage, the top 65% of the MobileNet backbone layers were unfrozen, and the learning rate was reduced to 0.000045. This two-stage approach ensures that the model precisely adapts to the specific visual characteristics of the customized dataset without destroying the generalized feature representations embedded in the pre-trained weights.

#### 4.3.3. Dataset

In order to train and validate the model, a combination of datasets specifically created for this work as well as public datasets has been used. In particular, 200 RGB photos with a resolution of 640 × 480 of the authorized person have been taken in various environments, lighting conditions, and positions. The photos were captured using the same camera employed for the prototype implementation, the OV5640, with features summarized in Table 3. Additionally, for the background, 600 photos of diverse backgrounds in rooms and offices were captured, also in RGB format and with a resolution of 630 × 480. Finally, the Human Faces dataset published on Kaggle [68] was used.

### 4.4. Algorithmic Complexity and Memory Footprint

The feasibility of deploying the proposed multimodal biometric system on a resource-constrained microcontroller (ESP32) relies on minimizing algorithmic complexity and memory operations. The network architectures were specifically selected and configured to reduce both temporal and spatial complexity.

For the person identification and facial authentication modules, the core computation is based on the MobileNetV2 architecture. As demonstrated in [69], convolutional operations are the primary source of computation and memory bandwidth in CNNs. Therefore, lower-order operations such as pooling, non-linear activations, or the final dense classification layer are asymptotically subsumed. The time complexity for a standard convolutional layer is mathematically defined as:

$$O(M^{2}\cdot K^{2}\cdot C_{in}\cdot C_{out})$$

where *M* is the spatial dimension of the output feature map, *K* is the kernel size, $C_{in}$ is the number of input channels, and $C_{out}$ is the number of output channels. By utilizing Depthwise Separable Convolutions, this complexity is strictly reduced to:

$$O(M^{2}\cdot K^{2}\cdot C_{in}+M^{2}\cdot C_{in}\cdot C_{out})$$

This factorization drastically decreases factorization drastically decreases the number of Multiply-Accumulate (MAC) operations. Furthermore, the facial biometric module employs a width multiplier of α=0.25. This hyperparameter proportionally thins the network, effectively decreasing the computational cost and the number of memory read/write operations by a quadratic factor of $\alpha^{2}\approx 0.0625$ (a 16-fold reduction) compared to the baseline model.

For the voice biometric module, the 1D Convolutional layers exhibit a reduced time complexity of:

$$O(M\cdot K\cdot C_{in}\cdot C_{out})$$

Given the small input dimensionality (13 MFCC coefficients) and the limited number of filters (maximum 32, as detailed in Table 6), the computational overhead is kept minimal. Regarding space complexity and memory operations O(S), the primary bottleneck in extreme edge devices is the SRAM required for storing intermediate activation maps during inference. The spatial complexity for the network weights is $O(K^{2}\cdot C_{in}\cdot C_{out})$ for standard layers, which translates directly to Flash memory usage, while the activation maps dictate volatile RAM usage. By applying post-training quantization (PTQ), the theoretical precision of weights and activations is reduced from 32-bit floating-point to 8-bit integers. As empirically demonstrated in the experimental evaluation (Section 5), this optimization significantly reduces memory bandwidth requirements, yielding a 40.18% reduction in RAM usage (down to just 6.4 KiB) and an 84.61% improvement in inference time. This ensures that the memory allocation operations remain strictly within the ESP32’s hardware limits while dramatically accelerating execution.

### 4.5. Threat Model and Security Considerations

While processing biometric data strictly at the extreme edge eliminates the vulnerabilities associated with cloud transmissions (e.g., Man in the Middle attacks, server-side data breaches, and unauthorized third-party data exploitation), there are other vulnerabilities associated with the end device itself. Local processing inherently maximizes user privacy by ensuring raw biometric captures (images and audio) never leave the device. However, it does not automatically guarantee full system security.

In our assumed threat model, we consider network-based attacks and remote data interception as the primary mitigated threats. In contrast, physical hardware attacks are considered out of scope for this study. Because the ESP32 microcontroller lacks advanced secure execution enclaves (such as strict TrustZone-like hardware isolation), the architecture relies on standard memory mapping. In our current proof-of-concept, the stored neural network weights and authorized user biometric templates (embeddings) reside in unencrypted Flash memory. Consequently, a highly motivated attacker with physical access to the device could theoretically dump the Flash memory via physical debug interfaces to extract these templates.

For a secure commercial deployment, the extreme edge architecture must be augmented with specific model and template protection mechanisms to prevent identity theft via inversion attacks. While traditional Homomorphic Encryption (HE) allows matching in the encrypted domain, it remains computationally prohibitive for highly constrained microcontrollers like the ESP32 [70]. Similarly, classic BioHashing provides a lightweight baseline but is vulnerable to combined attacks if the hardware secret is compromised [71].

Recent literature highlights deep-hashing and hybrid cancelable biometrics as the optimal solution for IoT edge devices [72]. These state-of-the-art approaches apply non-invertible, multivariate polynomial transformations to the embeddings, generating compact and revocable templates without significantly degrading the Equal Error Rate (EER) compared to floating-point baselines [73]. If the edge device is physically compromised, the template can be immediately revoked and regenerated, ensuring the user’s permanent biological trait remains uncompromised. For a production-ready system, these algorithmic protections should be coupled with the activation of the ESP32’s native Flash Encryption and Secure Boot features to prevent firmware readout. Implementing these joint mechanisms would ensure that both the private biometric templates and the system’s neural network topology remain strictly secured against physical extraction.

## 5. Results

The models described in Section 4 were integrated into a comprehensive multimodal biometric access control prototype. As previously detailed, the hardware deployment targeted the ESP32 development kit. To streamline the TinyMLOps workflow [26] and efficiently explore the quantization design space, the Edge Impulse platform was utilized.

While alternative literature often explores mathematical fusion techniques for multiple decisions, such as Borda count, highest rank, or logistic regression [49], our architecture deliberately implements a strict hierarchical filtering approach. This topology allows the system to forcefully “short-circuit” and abort the execution pipeline at the earliest sign of non-authorization. This strategy guarantees that the most computationally intensive models (facial biometrics) are only invoked when strictly necessary, thereby minimizing overall energy consumption. The aggregated hardware metrics of the complete hierarchical pipeline are summarized in Table 9. The entire end-to-end verification process executes in just 2719 ms on a highly constrained microcontroller, requiring a maximum RAM footprint of 377.3 KiB.

### 5.1. Person Detection Module Results

The evaluation outcomes for the preliminary person detection module are presented in Table 10 and Table 11. Post-Training Quantization (PTQ) techniques [74], specifically data quantization to 8-bit integers (INT8) and weight clustering, were applied utilizing the EON Tuner engine. This quantization yielded a massive 48.65% reduction in inference latency and decreased RAM consumption by 73.01%, making it viable for the ESP32.

While the isolated accuracy of this model (32.0% True Positive Rate) is the lowest among the implemented modules, it must be analyzed contextually as an energy-saving wake-up filter. Notably, the False Positive rate is exceptionally low (0.5%), guaranteeing that the system does not waste critical energy reserves waking up for empty frames. The 68.0% false negative rate is easily mitigated in a real-time operational stream; since a user naturally remains in the camera’s field of view over multiple consecutive frames, the cumulative probability of successfully detecting a cooperative user at least once across *n* frames is defined as $P\left(n\right)=1-{\left(FNR\right)}^{n}$. Applying our empirical FNR of 0.68, the detection probability rises steadily: 53.8% at n=2, 68.6% at n=3, 78.6% at n=4, and 90.1% by n=6. Given the 1.3-s inference latency of the FOMO module, the system mathematically guarantees a >90% probability of waking up within approximately 7.8 seconds of a user standing before the sensor, fully justifying its use as a low-power triggering mechanism.

**Figure 3:**
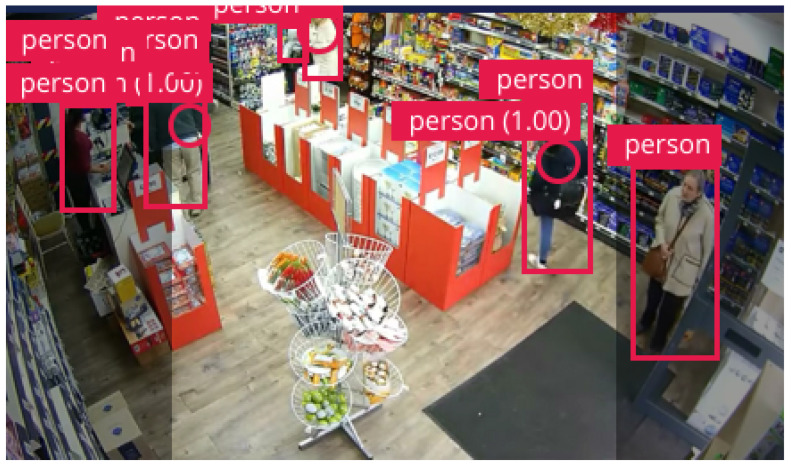
Classification errors.

Upon analyzing the failed predictions, two primary factors emerged. First, due to the severe downscaling of the input image, subjects situated on the far periphery occasionally fall outside the network’s effective receptive field. Second, as illustrated in Figure 3, when two individuals are positioned closely together within the same spatial grid, FOMO natively suppresses the secondary detection. Given that the prototype’s operational premise assumes a cooperative user standing directly in front of the access control device, these architectural constraints do not compromise the system’s practical reliability.

### 5.2. Voice Biometric Module Results

The empirical results for the voice biometrics module are summarized in Table 12 and Table 13. Following 8-bit quantization, the model maintained a robust overall F1-score while experiencing an 84.61% reduction in inference latency. Remarkably, the neural network inference executes in just 6 ms, utilizing merely 6.4 KiB of RAM. This highlights a critical paradigm shift in TinyML deployments: the computational bottleneck, in some designs, is no longer the neural network itself, but rather the Digital Signal Processing (DSP) feature extraction phase. In this implementation, calculating the MFCC coefficients introduces an estimated 200 ms of latency, dominating the execution time of the CNN.

Regarding classification performance, the “authorized” class exhibited a 91.3% True Positive rate, translating to a False Rejection Rate (FRR) of just 8.7%. Although this FRR implies that the system rejects the legitimate user roughly one in five times, it represents an acceptable practical compromise in extreme edge security systems. In these constrained environments, keeping the False Acceptance Rate (FAR) as low as possible (currently 6.25%) is prioritized over user convenience to prevent unauthorized access. Since a single voice inference requires only 296 ms (including the DSP phase) and consumes minimal energy, the practical cost of a false rejection is just a short delay for the user to repeat the passphrase. Furthermore, applying this threshold ensures that the subsequent, more energy-intensive facial recognition module is only activated when there is high confidence, which helps to optimize both overall system security and battery life.

### 5.3. Facial Biometric Module Results

The outcomes for the final facial authentication module are detailed in Table 14 and Table 15. The impact of INT8 quantization is particularly significant in this module. The unoptimized 32-bit float model required a prohibitive 7546 ms to execute on the ESP32. Quantization reduced this inference time to 1102 ms (an 85.4% reduction), bringing the latency within acceptable operational parameters for a smart door access scenario while halving the RAM footprint.

### 5.4. Hierarchical System Evaluation

To provide a clearer perspective on the system’s security and usability, the standard biometric metrics False Acceptance Rate (FAR) and False Rejection Rate (FRR) were extracted from the operational confusion matrices. As shown in Table 16, both modules prioritize security over convenience, maintaining a strict, low FAR. While the FRR values indicate that the authorized user might occasionally need a second attempt to authenticate, the low FAR (4.7% for voice and 3.3% for facial) ensures robust protection against unauthorized access attempts.

In biometric access control systems, standard accuracy is often a misleading metric due to class imbalance; therefore, we evaluate performance strictly through FAR and FRR. To fully assess the operational security of the prototype, it is crucial to evaluate the end-to-end multimodal performance. Because the system employs a serial hierarchical architecture (the facial recognition module is only triggered if the voice module grants initial authorization), the combined system metrics can be mathematically modeled as an AND-rule fusion.

The global False Acceptance Rate ($FAR_{sys}$) represents the probability of an impostor successfully bypassing both the voice and facial filters consecutively. It is calculated as:

(3) $$FAR_{sys}=FAR_{voice}\times FAR_{face}=0.0625\times 0.019\approx 0.00118$$

This multiplicative effect reduces the system’s overall FAR to 0.12%. This demonstrates that the serial pipeline offers a promising security proposal against unauthorized access.

Conversely, the global False Rejection Rate ($FRR_{sys}$) represents the probability that a legitimate user is rejected by either of the modules:

(4) $$FRR_{sys}=1-\left(\left(1-FRR_{voice}\right)\times \left(1-FRR_{face}\right)\right)=1-\left(0.913\times 0.838\right)\approx 0.2349$$

While an overall FRR of 23.49% indicates that the authorized user may need to repeat the authentication process roughly one in three attempts, this is a deliberate and acceptable trade-off. As discussed earlier, in severely constrained environments where absolute security is prioritized, maintaining a FAR of 0.12% outweighs the minor inconvenience of a repeated 2.7-s inference cycle.

### 5.5. Energy Consumption and Viability

For the purpose of energy measurements, an XDM2041 bench multimeter was employed. To obtain accurate results, measurements were repeated 10 times, with the maximum current and voltage values averaged. As detailed in Table 9, the energy consumed during a single, complete multimodal inference ($E_{inference}$) is the sum of the energy consumed by the three individual biometric filters, see Equation (5).

(5) $$\begin{array}{c} E_{inference}=E_{person}+E_{facial}+E_{voice}=0.068\;J+0.069\;J+0.017\;J=0.154\;J \end{array}$$

Assuming a standard battery with a capacity of 600 mAh at 3.7 V, the total available energy is $E_{battery}=7992\;J$. If the system were to run continuously without interruption, it could operate for an average of approximately 38.8 h.

However, this continuous operation estimation represents a theoretical worst-case baseline. In a practical deployment, the system leverages an interrupt-driven architecture triggered by a low-power PIR sensor, keeping the microcontroller in deep sleep mode ($I_{sleep}\approx 10\;\mu A$) the majority of the time. To provide a realistic assessment of the device’s autonomy, we define the daily energy consumption $E_{day}\left(N\right)$ as a function of the number of daily access attempts *N* as can be seen in Equation (6).

(6) $$\begin{array}{c} E_{day}\left(N\right)=\left(E_{sleep\_day}+N\cdot E_{inference}\right)\cdot \sigma \end{array}$$

where $E_{sleep\_day}$ is the energy consumed during 24 h of deep sleep (3.19J), and σ=1.2 is a safety margin factor to account for sensor initialization, wake-up overhead, and voltage conversion losses. Consequently, the estimated battery life in days is calculated as $T\left(N\right)=E_{battery}/E_{day}\left(N\right)$.

As shown in Table 17, for a typical office or laboratory environment with 50 to 100 daily accesses, the system can operate autonomously for approximately one year. This demonstrates that the proposed TinyML architecture is highly efficient and represents a viable baseline for long-term deployments in energy-constrained IoT environments.

## 6. Discussion

The results obtained in this study underscore a fundamental paradigm shift in the deployment of intelligent sensors: moving from cloud-based and high-performance edge gateways toward extreme edge computing (TinyML). While the theoretical advantages of multimodal biometrics, such as overcoming unimodal vulnerabilities and spoofing attacks, have been well documented since the foundational work of Ross et al. [39], the physical implementation of these systems has historically remained a bottleneck for IoT scalability.

A critical observation from our comparative analysis (Table 1) is the widespread hardware disparity in the literature. Many recent studies addressing “mobile” or “edge” biometrics, such as the multimodal systems proposed by Zhang et al. [75], Kocacinar et al. [40], and Huang et al. [41], rely on high-tier smart terminals or smartphones. While these devices operate locally, they possess gigabytes of RAM and multi-core application processors. In contrast, our proposed architecture is executed entirely on a commercial off-the-shelf (COTS) ESP32 microcontroller, consuming a maximum of 377.3 KiB of RAM and 157.5 KiB of Flash memory. This demonstrates that robust biometric authentication does not require complex operating systems or massive computational power, making it economically and physically viable for ubiquitous, low-cost smart environments.

Regarding algorithmic performance, there is an inherent trade-off between the metrics achieved by server-grade deep learning models and the extreme constraints of TinyML. Studies such as Alharbi and Alshanbari [46] and Al-Waisy et al. [49] utilize heavy architectures like FaceNet or deep CNNs to achieve near-perfect performance across all metrics. In contrast, our heavily quantized (INT8) models are deliberately designed to prioritize security over user convenience. By accepting a moderate False Rejection Rate (FRR), the serial pipeline achieves an exceptionally stringent combined False Acceptance Rate (FAR) of just 0.12%. In the context of localized access control, requiring a user to occasionally repeat an authentication attempt is a highly acceptable trade-off for a system that requires only 0.154 J per inference cycle and guarantees absolute data privacy by never transmitting raw biometric features over a network.

Furthermore, the integration strategy plays a vital role in energy efficiency. Traditional multimodal systems frequently employ score-level or feature-level fusion to maximize accuracy [46,47]. However, evaluating all modalities simultaneously is highly energy-intensive. Our architecture deliberately utilizes a hierarchical, early-exit filtering strategy. By employing an ultra-lightweight person-detection filter (FOMO) as an intelligent wake-up mechanism, the system effectively short-circuits the processing pipeline. The most computationally expensive module (facial recognition, requiring 1102 ms) is only invoked if both human presence and preliminary voice authorization are confirmed.

Moreover, while custom Application-Specific Integrated Circuits (ASICs), such as those explored by Vitolo et al. [44], offer superior micro-joule efficiency for specific tasks like keyword spotting, our software-based TinyML approach provides unparalleled flexibility. Utilizing tools like Edge Impulse to optimize standard neural networks for general-purpose microcontrollers enables rapid prototyping and updating of smart sensors without the prohibitive costs of custom silicon manufacturing.

Regarding environmental robustness, the system mitigates acoustic interference through Gaussian noise injection during the voice model’s training phase, successfully filtering ambient clutter. Visually, the reliance on a pre-trained MobileNetV2 backbone endows the facial authentication module with inherent resilience to ambient lighting variations and background shifts, a characteristic derived from its extensive large-scale pre-training.

Finally, a critical consideration for the real-world deployment of this system is its robustness against Presentation Attacks (PAs), commonly referred to as spoofing. In its current iteration, the prototype relies on standard 2D RGB imaging and PCM audio, making it theoretically vulnerable to replay attacks (e.g., high-fidelity audio recordings) or printed photograph attacks. Implementing software-based liveness detection or Presentation Attack Detection (PAD), such as evaluating micro-expressions, blink detection, or lip-syncing would exceed the remaining computational and memory budget of the ESP32. Therefore, to achieve commercial-grade spoofing immunity without compromising the extreme-edge processing latency, future hardware iterations should substitute the standard CMOS camera with multi-spectral or depth-sensing modules (e.g., Infrared or Time-of-Flight sensors). These hardware-assisted PAD mechanisms can verify liveness prior to triggering the neural network inference, maintaining the system’s strict energy efficiency.

## 7. Conclusions

This work demonstrates the feasibility of deploying standalone, multimodal biometric authentication systems at the extreme edge. By executing complex machine learning algorithms directly on the sensing node, the proposed solution provides a robust framework for “privacy by design”, eliminating the reliance on external cloud servers and network connectivity. The hierarchical filtering architecture prioritized security, achieving a stringent False Acceptance Rate (FAR) of 6.25% for voice and 1.9% for facial verification. When combined in series, the system-level FAR drops to 0.12%. Demonstrating promising energy efficiency, the prototype can sustain continuous, uninterrupted inference for nearly 39 h on a 600 mAh battery. Under realistic access-control duty cycles utilizing the microcontroller’s deep-sleep modes, this translates to several months of autonomous operation on a single charge.

Despite its success, the current system presents certain limitations regarding environmental benchmarking, which define critical future research directions. While the system was evaluated under realistic laboratory conditions and successfully mitigated standard background noise, comprehensive longitudinal testing (repeated sessions over extended periods to account for template aging) and extreme variance in user pose and illumination were outside the scope of this study. Furthermore, the reliance on localized “few-shot” dataset enrollment, while highly realistic for user onboarding, inherently limits the generalization capabilities of the models compared to those trained on massive, varied datasets. Future work will explore the implementation of lightweight Siamese networks for robust one-shot learning at the edge. Additionally, the spatial data inherent in the FOMO person-detection module could be utilized to dynamically guide motorized camera mounts, actively mitigating pose and distance variations by positioning the sensor for optimal facial capture before the final inference stage is executed.

To the best of our knowledge, this study presents the first comprehensive implementation of a hierarchical, multimodal TinyML biometric pipeline on a strictly resource-constrained microcontroller such as the ESP32 or equivalent. The development process highlights the importance of hardware–software co-design. Utilizing built-in microcontroller features, such as vector extensions, domain-specific architectures, and optimized DSP libraries, is essential for running complex neural networks in edge environments. Ultimately, this research demonstrates how TinyML can transform traditional embedded systems into secure, energy-efficient smart sensors, enabling privacy-preserving IoT ecosystems.

## Figures and Tables

**Table 1:** Performance and hardware comparison between traditional deep learning-based recognition systems and the proposed resource-constrained hierarchical TinyML pipeline.

Ref.	Task	Precision	Latency	Resources	Device	Method
[40]	Mask detection	90.4%	N/A	N/A	Android Smart Phone	CNN
[41]	Object recognition	99.82%	70 ms	N/A	Huawei P30	YOLO, SSD
[43]	Voice recognition	89%	214–213 ms	N/A	Android Smart Phone	MFCC + GMM
[50]	Iris, periocular, palmprint, face recognition	99.02%, 100%, 100%, 100%	N/A	N/A	Intel Core i5	SVM
[48]	Iris recognition	89–100%	60–90 ms	N/A	Intel Core i7	CNN + SVM
[49]	Iris recognition	99.82%	620 ms	N/A	Intel Xeon E5-1620 CPU	CNN + Ranking Level fusion
[51]	Face recognition	95.84–99.19%	N/A	N/A	N/A	Tree of CNNs
Ours	Person, Voice, Face recognition	32% (Person), System FAR 0.12%	1321 ms, 296 ms, 1102 ms	78.2 KiB, 52.1 KiB, 27.2 KiB	ESP32	FOMO, MFCC + CNN, CNN

Note: N/A = Not Available.

**Table 2:** Evaluated hardware specifications.

MCU	Processor	RAM	Flash	Price	Connectivity
ESP-WROVER-KIT V3, ESP32, (Espressif Systems, Shanghai, China)	2 cores Xtensa 32-bit LX6	4 MB	4 MB	33.75 €	I/O, JTAG, USB, Camera, UART, SPI, Micro-SD, 2.4 GHz Wi-Fi and Bluetooth LE
Arduino Nano 33 (nRF52840) (Arduino, Turin, Italy)	Arm Cortex-M4F (with FPU)	256 KB	1 MB	35.10 €	I/O, ADC, USB, UART, SPI, I2C, Bluetooth and Wi-Fi
Raspberry Pi Pico RP2040 (Raspberry Pi Foundation, Cambridge, UK)	Dual-core ARM Cortex-M0+	264 KB	2 MB	11.50 €	I/O, ADC, USB, Wi-Fi and Bluetooth, UART, SPI, I2C, PWM

**Table 3:** OV5640 specifications.

**CMOS Sensor**	OmniVision OV5640
**Active Pixels**	2592 (H) × 1944 (V) = 5MP
**Pixel Size**	1.4 μm × 1.4 μm
**Optical Format**	1/4″ (Diagonal 4.6 mm)
**Shutter Type**	Rolling Shutter
**Chromaticity**	Color
**Illuminated type**	Back Side Illuminated (BSI)
**Maximum Frame Rate (UYVY)**	2592 × 1944 @ 15 fps 1920 × 1080 @ 30 fps 1280 × 720 @ 60 fps 640 × 480 @ 30 fps
**Maximum S/N Ratio**	36 dB
**Input Clock Range**	48 MHz
**Module Data Transmission**	MIPI CSI-2, up to 2 lanes
**Power Consumption**	2592 × 1944 @ 15 fps 294 mW
**Standby Power**	18 mW Standby

**Table 4:** Performance comparison of FOMO and YOLO [58].

Model	Epochs	Input Size	Performance
YOLOv8	20	320 × 320	84.9% mAP
YOLOv5	20	320 × 320	64.2% mAP
FOMO	20	320 × 320	80% F1 score

**Table 5:** MP34DT05 specifications.

Feature	Description
Output format	PDM
Sensitivity FoV	Omnidirectional
Sensitivity	−26 dBFS ± 3 dB
SNR	64 dB
AoP	dBSPL

**Table 6:** MFCC coefficients.

Parameter	Value
Coefficients	13
Length	0.02
Stride	0.02
# filters	32
FFT length	256
Window size	101
Min Freq (Hz)	0
Max Freq (Hz)	8000
Pre Coef	0.98

**Table 7:** Voice biometric neuronal network architecture.

Layer	# Filters	Size	p	s	Func
Gaussian Noise	-	-	-	-	-
Reshape	-	-	-	-	-
Conv1D	8	3	same	-	relu
MaxPooling1D	-	-	same	2	-
Dropout	-	-	-	-	-
Conv1D	16	3	same	-	relu
MaxPooling1D	-	-	same	2	-
Dropout	-	-	-	-	-
Flatten	-	-	-	-	-
Dense (y_pred)	3	-	-	-	softmax

**Table 8:** Facial Biometric recognition network architecture.

Layer/Block	Output Shape	Parameters	Trainable
Input Image	(96, 96, 3)	-	-
MobileNet Base (α=0.25)	Multiple	Pre-trained	Partially (Fine-tuned)
Reshape	(−1, Feature Map)	0	-
Dropout (p=0.1)	-	0	-
Flatten	-	0	-
Dense (Softmax)	(3)	Custom	Yes

**Table 9:** Global biometric pipeline specifications.

Parameter	Person	Facial	Voice	Total
Inference Time (ms)	1321	1102	296	2719
RAM Usage (KiB)	239.4	134.2	3.7	377.3
Flash (KiB)	78.2	52.1	27.2	157.5
Power (W)	0.0520	0.0628	0.0594	-
Energy (J)	0.068	0.069	0.017	0.154

**Table 10:** Person detection before and after quantization comparison.

Parameter	Original	Quantized	Improvement (%)
Inference Time (ms)	2573	1321	48.65
Ram Usage (KiB)	887.1	239.4	73.01
Flash (KiB)	101.6	78.2	23.03

**Table 11:** Person detection confusion matrix after model quantization. Green cells indicate correct classifications, while light red cells represent misclassifications.

Actual/Predicted	Background	Person
Background	99.5%	0.5%
Person	68.0%	32.0%
f1 score	0.99	0.45

**Table 12:** Voice biometric: Before and after quantization comparison.

Parameter	Float32 (Original)	INT8 (Quantized)	Improvement (%)
Inference Time (ms)	39	6	84.61
RAM Usage (KiB)	10.7	6.4	40.18
Flash (KiB)	54.8	52.1	4.92

**Table 13:** Voice biometric: INT8 Confusion Matrix. Green cells indicate correct classifications, while light red cells represent misclassifications.

Actual/Predicted	Authorized	Background	Unknown
Authorized	91.3%	0%	8.7%
Background	0%	93.2%	6.8%
Unknown	6.25%	1.56%	92.19%
f1 score	0.87	0.95	0.66

**Table 14:** Facial Authentication: Before and after quantization comparison.

Parameter	Float32 (Original)	INT8 (Quantized)	Improvement (%)
Inference Time (ms)	7546	1102	85.40
Ram Usage (KiB)	274.3	134.2	51.08
Flash (KiB)	870.5	318.1	63.46

**Table 15:** Facial Authentication: INT8 Confusion Matrix. Green cells indicate correct classifications, while light red cells represent misclassifications.

Actual/Predicted	Background	Authorized	Unknown
Background	91.4%	1.6%	6.9%
Authorized	0%	83.8%	16.2%
Unknown	1.9%	1.9%	96.1%
f1 score	0.95	0.86	0.88

**Table 16:** End-to-end operational metrics for the multimodal biometric system.

Modality	FAR	FRR
Voice Biometric (INT8)	6.25%	8.7%
Facial Biometric (INT8)	1.9%	16.2%
Hierarchical System	0.12%	23.49%

**Table 17:** Estimated battery life under different realistic duty-cycle scenarios (600 mAh battery).

Accesses/Day (*N*)	Daily Energy (J)	Autonomy (Days)	Autonomy (Months)
10	5.68	1406	∼46.2
50	13.08	611	∼20.1
100	22.32	358	∼11.8
500	96.24	83	∼2.7

## Data Availability

The data presented in this study are available on request from the corresponding author. The data are not publicly available due to privacy restrictions.
